# Stat3 modulates chloride channel accessory protein expression in normal and neoplastic mammary tissue

**DOI:** 10.1038/cddis.2016.302

**Published:** 2016-10-06

**Authors:** Katherine Hughes, Maximilian Blanck, Sara Pensa, Christine J Watson

**Affiliations:** 1 Department of Veterinary Medicine, University of Cambridge, Madingley Road, Cambridge CB3 0ES, UK; 2 Department of Pathology, University of Cambridge, Tennis Court Road, Cambridge CB2 1QP, UK

## Abstract

Mammary gland regression at the cessation of lactation (involution) is an exquisitely orchestrated process of cell death and tissue remodelling in which Stat3 signalling has an essential role. The involution microenvironment of the mammary gland is considered to be pro-tumourigenic and a proportion of cases of pregnancy-associated breast cancer are suggested to originate in tandem with involution. However, the apparent paradox that STAT3 is required for cell death in normal mammary gland, but is associated with breast cancer cell survival, has not been resolved. Herein, we investigate Stat3-mediated regulation of expression of members of the calcium-activated chloride channel regulator (CLCA) family of proteins during involution and mammary carcinogenesis. Using the conditionally immortal mammary epithelial cell line KIM-2, together with mice exhibiting mammary epithelial cell-specific deletion of Stat3 during lactation, we demonstrate that expression of mCLCA1 and mCLCA2 is elevated in concert with activation of Stat3. By contrast, murine CLCA5 (mCLCA5), the murine orthologue of human CLCA2, is significantly upregulated at 24, 72 and 96 h of involution in Stat3 knockout mice, suggesting a reciprocal regulation of these proteins by Stat3 *in vivo*. Interestingly, orthotopic tumours arising from transplantation of 4T1 murine mammary tumour cells exhibit both phosphorylated Stat3 and mCLCA5 expression. However, we demonstrate that expression is highly compartmentalized to distinct subpopulations of cells, and that Stat3 retains a suppressive effect on mCLCA5 expression in 4T1 tumour cells. These findings enhance our understanding of the regulation of CLCA channel expression both *in vitro* and *in vivo*, and in particular, demonstrate that expression of mCLCA1 and mCLCA2 during involution is profoundly dependent upon Stat3, whereas the relationship between mCLCA5 and Stat3 activity is reciprocal and restricted to different subpopulations of cells.

At the cessation of lactation, the two-phase process of mammary gland involution restores the organ to near its pre-pregnant state, and is exquisitely regulated by factors including transforming growth factor-*β*3 during the first phase of involution,^1^ IL-6 cytokine family members leukaemia inhibitory factor (LIF) (first phase)^2, 3^ and oncostatin M (OSM) (second phase).^4^ These cytokines activate Stat3, a critical transcriptional coordinator of the involution process.^5, 6^ Thus, mice with a mammary-specific conditional deletion of Stat3 exhibit markedly impaired involution and diminished cell death.^7, 8, 9, 10^

Although the initial phase of involution is reversible, the second irreversible stage incorporates further cell death, extensive tissue remodelling and acquisition of a ‘wound-healing phenotype'.^11^ The inflammatory milieu of the involution mammary gland is considered to be pro-tumourigenic, illustrated by mouse models of involution-associated tumourigenesis.^12, 13^ A corollary of these observations may be found in epidemiological evidence from human breast cancer indicating not only that a subgroup of cases of the wider set of ‘pregnancy-associated breast cancers' arises in the post-partum period, but that this subset of patients may have a poorer prognosis.^14^

Chloride channel accessory proteins, also known as calcium-activated chloride channel regulators (CLCA proteins), are a family of transmembrane proteins that have been suggested to have a role in chloride conductance in epithelial cells. Although the mechanism by which they activate other channel proteins to trigger movement of chloride ions across membranes is not clear, there is some evidence that they can be secreted, and thereby potentially activate chloride channels.^15^

CLCA proteins have been associated with diverse functions including cell adhesion,^16^ apoptosis, tumourigenesis and mucus cell differentiation.^17^ Of particular interest, both CLCA2 and CLCA4 have been shown to inhibit proliferation of breast cancer cells when ectopically expressed *in vitro*,^18^ whereas normal breast tissue exhibits high levels of CLCA2 in both acini and small ducts.^19^ Murine CLCA5 (mCLCA5) is highly homologous to human CLCA2 (hCLCA2) and is considered to be its orthologue.^20^ Widely expressed in the cytoplasmic granules of granular layer keratinocytes in stratified squamous epithelia,^21^ mCLCA5 is downregulated in some murine mammary tumour cell lines, including 4T1 cells, where re-expression inhibits proliferation.^22^ Thus, hCLCA2 and its murine orthologue have been proposed as tumour suppressor genes in breast cancer.^23, 24^ However, other investigators have demonstrated an upregulation of hCLCA2 in triple-negative breast cancer patients with a poor prognosis,^25^ perhaps suggesting breast cancer subtype specific roles, and exemplifying the need for further characterization of this poorly understood protein family.

Other murine genes include *mClca1* and *mClca2* that share ~96% complementary DNA sequence identity, and encode mCLCA1 and mCLCA2, respectively.^26^ These proteins are reciprocally expressed during mammary gland development with mCLCA2 being expressed at high levels during lactation and involution, whereas mCLCA1 expression is suppressed during involution.^27^ This pattern suggests different functional roles for these highly related genes.

Given the pro-tumourigenic potential of the involution mammary microenvironment, the critical role of Stat3 in orchestrating regression and the intriguing regulation of mCLCA proteins, we hypothesized that mammary epithelial expression of Stat3 would influence the expression of mCLCA proteins.

## Results

### Expression of mCLCA2 and mCLCA5 in mammary epithelial KIM-2 cells is modulated by Stat3 activity

**Figure 1:**
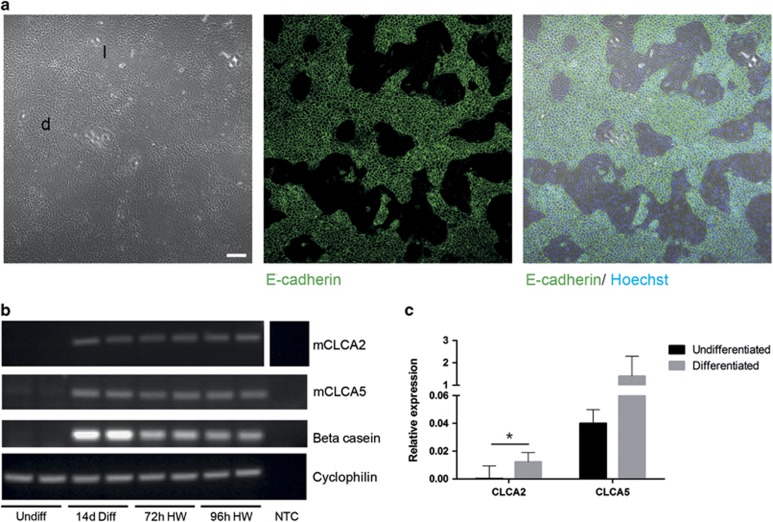
KIM-2 mammary epithelial cells exhibit upregulation of mCLCA2 and mCLCA5 expression upon differentiation, which persists during hormone withdrawal. (**a**) Upon stimulation with prolactin, KIM-2 cells form dome-like epithelial islands (d), which exhibit robust E-cadherin expression, surrounded by more elongate, loosely arranged cells (l). Immunofluorescence staining for E-cadherin (green) and DNA (Hoechst; blue). Right panel shows merged images. Scale bar=100 *μ*m. (**b**) RT-PCR analysis for mCLCA2, mCLCA5, *β*-casein and cyclophilin A (housekeeping gene), in KIM-2 cells that are undifferentiated (undiff), KIM-2 cells that have received lactogenic hormones for 14 days (14d Diff), or KIM-2 cells that have received lactogenic hormones for 11–14 days and have then undergone hormone withdrawal for 72 (72 h HW) or 96 h (96 h HW). Two independent biological repeats of each condition are shown. (**c**) Expression of mCLCA2 and mCLCA5 in undifferentiated and differentiated KIM-2 cells, measured by qRT-PCR relative to expression of cyclophilin A (housekeeping gene); values are mean±S.D. from at least three biological repeats; *P<0.05, as determined by Student's *t*-test. NTC, no template control

To interrogate the hypothesis that mammary epithelial expression of Stat3 will influence the expression of mCLCA proteins, we first wished to establish baseline levels of expression of mCLCA2 and mCLCA5 in the KIM-2 conditionally immortal mouse mammary epithelial cell line. KIM-2 cells were maintained in an undifferentiated state and were subsequently differentiated by addition of lactogenic hormones, resulting in the emergence of two phenotypically distinct populations – an epithelial component expressing E-cadherin, and forming distinct islands and dome-like structures, and a more loosely arranged population of elongated cells around these islands (Figure 1a), as previously described.^28^ Differentiation was confirmed by robust upregulation in expression of the milk protein *β*-casein (Figure 1b). Interestingly, both mCLCA2 and mCLCA5 exhibited a pronounced upregulation in expression upon differentiation, with mCLCA5 exhibiting higher levels of expression than mCLCA2. This elevated expression persisted during the first 96 h of hormone withdrawal, a situation that mimicks involution (Figure 1).

**Figure 2:**
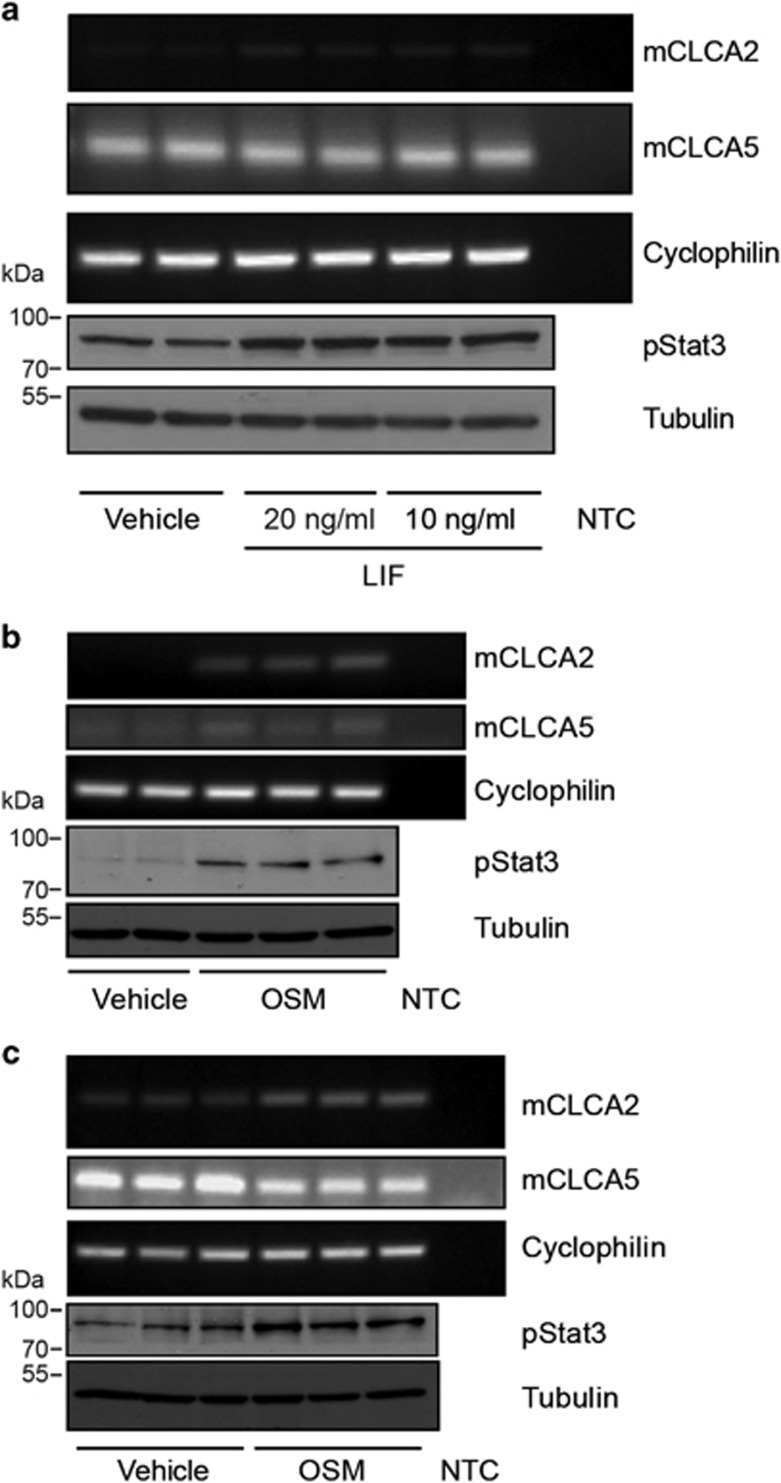
Undifferentiated mammary epithelial KIM-2 cells exhibit low levels of expression of mCLCA2 and mCLCA5, and expression of mCLCA2 may be upregulated upon stimulation with LIF or OSM. Differentiated KIM-2 cells upregulate expression of mCLCA2 upon stimulation with OSM and downregulate expression of mCLCA5. (**a**) RT-PCR analysis for mCLCA2, mCLCA5 and cyclophilin A (housekeeping gene), and western blot analysis of phosphorylated Stat3 (pStat3) and tubulin expression in undifferentiated KIM-2 cells stimulated with either vehicle or 10 or 20 ng/ml LIF. Two biological repeats are shown. (**b**) RT-PCR analysis for mCLCA2, mCLCA5 and cyclophilin A, and western blot analysis of pStat3 and tubulin expression in undifferentiated KIM-2 cells stimulated with either vehicle or 25 ng/ml OSM. Two (vehicle) or three (OSM stimulated) biological repeats are shown. (**c**) RT-PCR analysis for mCLCA2, mCLCA5 and cyclophilin A, and western blot analysis of pStat3 and tubulin expression in differentiated KIM-2 cells stimulated with either vehicle or 25 ng/ml OSM. Three biological repeats are shown. NTC, no template control

Given the striking upregulation of mCLCA2 and mCLCA5 upon differentiation of the KIM-2 cells and during the involution-like process precipitated by hormone withdrawal, we wished to investigate the impact of Stat3 signalling. Undifferentiated KIM-2 mammary epithelial cells were stimulated with either LIF or OSM to activate Stat3 by phosphorylation. Basal levels of expression of mCLCA2 and mCLCA5 were low/undetectable in undifferentiated KIM-2 cells (Figures 1b, 2a and b), but a modest increase in expression of mCLCA2 was seen upon stimulation with both LIF (Figure 2a) and OSM (Figure 2b) mirroring the upregulation in phosphorylated Stat3 (pStat3). Modulation of mCLCA5 expression was not observed. As anticipated, expression of mCLCA2 was also enhanced by stimulation of differentiated KIM-2 cells with OSM, but intriguingly this resulted in a modest decrease in mCLCA5 expression (Figure 2c), possibly reflecting differences in receptor downstream signalling pathways in differentiated cells. This was also reflected in the response of differentiated KIM-2 cells to LIF where upregulation of pStat3 was not observed (Supplementary Figure 1), despite continued expression of the LIF receptor at the RNA level (data not shown).

These findings suggest that two of the key upstream regulators of Stat3 during involution, namely LIF and OSM, regulate CLCA expression in murine mammary epithelium, possibly in a reciprocal manner, with mCLCA2 being induced, while mCLCA5 is suppressed. This result prompted us to investigate CLCA expression *in vivo*.

### mCLCA1 and mCLCA2 are upregulated, and mCLCA5 is downregulated, during involution in a Stat3-dependent manner

**Figure 3:**
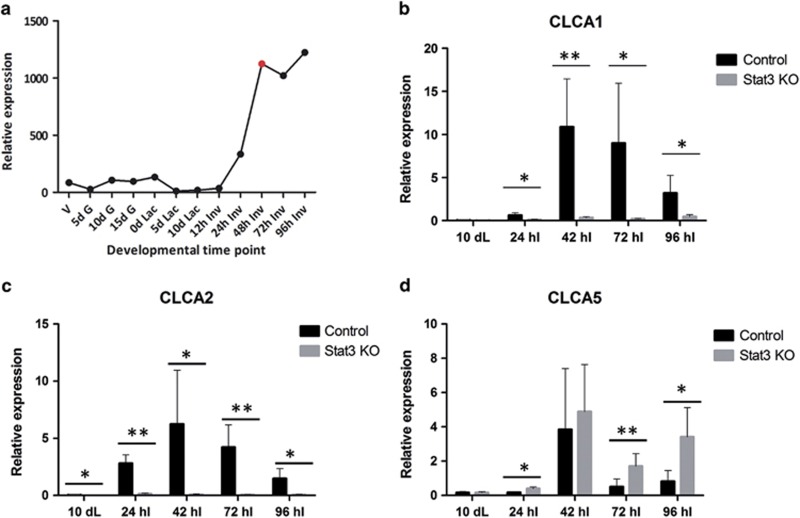
mCLCA1 and mCLCA2 are profoundly upregulated during involution in a Stat3-dependent manner. By contrast, mCLCA5 is upregulated in the absence of epithelial Stat3 signalling. (**a**) Microarray expression profile for mCLCA1 derived from 12 different time points in the developmental cycle of the mouse mammary gland. Expression of mCLCA1 (**b**), mCLCA2 (**c**) and mCLCA5 (**d**) in control and Stat3 KO mice measured by qRT-PCR relative to expression of cyclophilin A (housekeeping gene). Values are mean±S.D. from at least three biological repeats; *P<0.05, **P<0.01 as determined by Student's *t*-test. d G, days gestation; dL, days lactation; d Lac, days lactation; hI, hours involution; h Inv, hours involution; V, virgin

Affymetrix (High Wycombe, UK) microarray data that have been previously published,^29^ indicated that mCLCA3 is expressed at very low levels (data not shown), whereas expression of mCLCA1 is markedly upregulated between 12 and 48 h of involution (first phase), and subsequently maintained from 48 h onwards (conventionally considered the onset of the murine irreversible second phase; Figure 3a).

To further interrogate a possible relationship between Stat3 activity and CLCA protein expression, we utilized mice with a mammary epithelial cell-specific conditional deletion of Stat3 (*Stat3*^*fl/fl*^; *BLG-Cre*; hereafter referred to as Stat3 KO) and compared these with age-matched controls lacking Cre expression (control). Using qRT-PCR, we compared expression of mCLCA1, mCLCA2 and mCLCA5 during lactation and involution, utilizing samples collected at 42 h involution, close to the onset of the irreversible phase (unpublished data).

mCLCA1, mCLCA2 and mCLCA5 are all significantly upregulated during involution, particularly, at the onset of the second phase. Strikingly, expression of mCLCA1 and mCLCA2 is almost entirely abrogated in the absence of epithelial Stat3 (Figures 3b and c). Conversely, mCLCA5 expression exhibits a different pattern of expression reaching maximal levels at the 42 h of involution ‘switch point' to irreversibility, and thereafter declining. Interestingly, expression is not abrogated by deletion of Stat3, and is instead significantly upregulated in the Stat3 KO mice at 24, 72 and 96 h of involution (Figure 3d). This correlates with the observation that mCLCA5 is downregulated upon stimulation of Stat3 activity in OSM-stimulated differentiated KIM-2 cells (Figure 2c).

**Figure 4:**
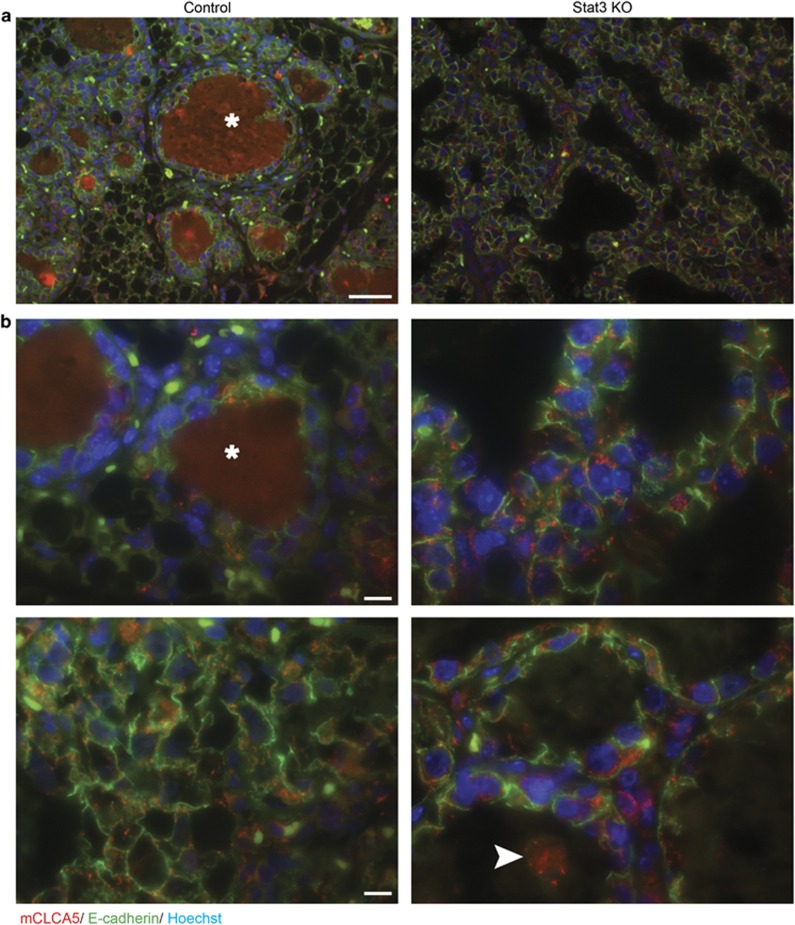
mCLCA5 protein is expressed during mammary gland involution and is subjectively more abundant in the absence of epithelial Stat3 signalling. Immunofluorescence staining for mCLCA5 (red) and E-cadherin (green), and DNA (Hoechst; blue) on control and Stat3 KO tissue at 96 h of involution. Scale bar=50 *μ*m (**a**) and 10 *μ*m (**b**). Staining of residual milk (*) was considered non-specific. Some, but not all shed cells exhibited positive staining for mCLCA5 (arrowhead). Representative images are displayed. Sections from five mice were examined (control *n*=2; Stat3 KO *n*=3)

At 96 h of involution, modest expression of mCLCA5 is detectable in mammary epithelial cells in both control and Stat3 KO mice (Figure 4a and Supplementary Figure 2A). In control animals, expression of mCLCA5 is multifocally variable in its intensity, which may reflect differing levels of mammary alveolar regression within the gland at this time (Figure 4b). Correlating with the qRT-PCR data (Figure 3d), there is apparent increased expression of mCLCA5 in the absence of epithelial Stat3 signalling (Figure 4b and Supplementary Figure 2B). Intriguingly, mCLCA5 is present in some intraluminal shed (dying) epithelial cells, although this is not a uniform finding.

### mCLCA5 and Stat3 may be reciprocally expressed during tumourigenesis

We considered our observations in the Stat3 KO mice to be highly relevant, given that constitutively active Stat3 signalling is observed in approximately half of primary breast cancers^30^ and that mCLCA5 is the murine orthologue of the human putative tumour suppressor hCLCA2, as previously discussed.^20^ It therefore appeared logical that Stat3 signalling in tumours may have an inhibitory effect upon expression of mCLCA5 and thus we explored this hypothesis in the 4T1 murine syngeneic tumour model, which has previously been reported to exhibit minimal expression of mCLCA5.^22^

**Figure 5:**
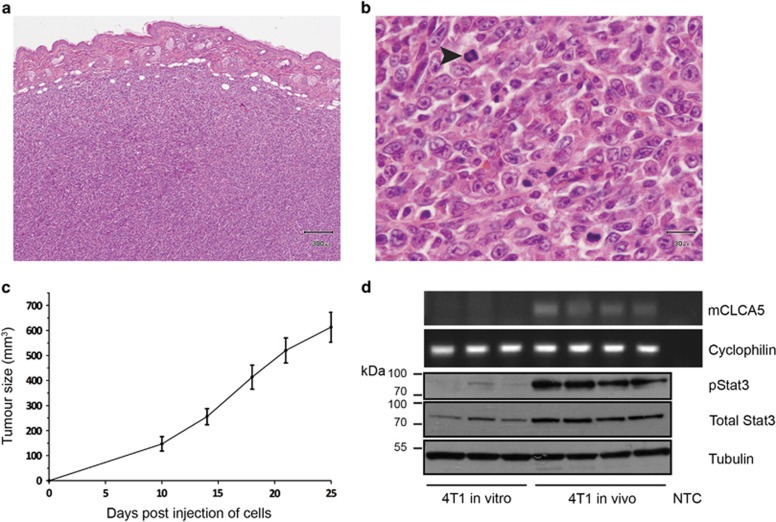
mCLCA5 is expressed in tumours resulting from implantation of 4T1 cells. (**a** and **b**) Representative photomicrographs demonstrating that orthotopic tumours derived from 4T1 cells are densely cellular, with minimal stroma and a moderately to markedly pleomorphic phenotype with numerous mitoses. Haematoxylin and eosin stain. Scale bar=300 *μ*m (**a**) and 30 *μ*m (**b**). Arrowhead indicates a mitotic figure. Several mitoses are present in this field. (**c**) Average tumour size in mm^3^ for tumours derived from injection of 1 × 10^5^ 4T1 cells in the mammary fat pad. Values are mean±S.D. from four biological repeats. (**d**) RT-PCR analysis for mCLCA5 and cyclophilin A, and western blot analysis of phosphorylated Stat3 (pStat3), total Stat3 and tubulin expression in 4T1 cells maintained in culture (4T1 *in vitro*) and in tumours resulting from implantation of 4T1 cells into the mammary fat pad of syngeneic mice (4T1 *in vivo*). Three independent biological repeats are shown for 4T1 *in vitro*; for 4T1 implanted in mice, four tumours from separate individuals are represented. NTC, no template control

**Figure 6:**
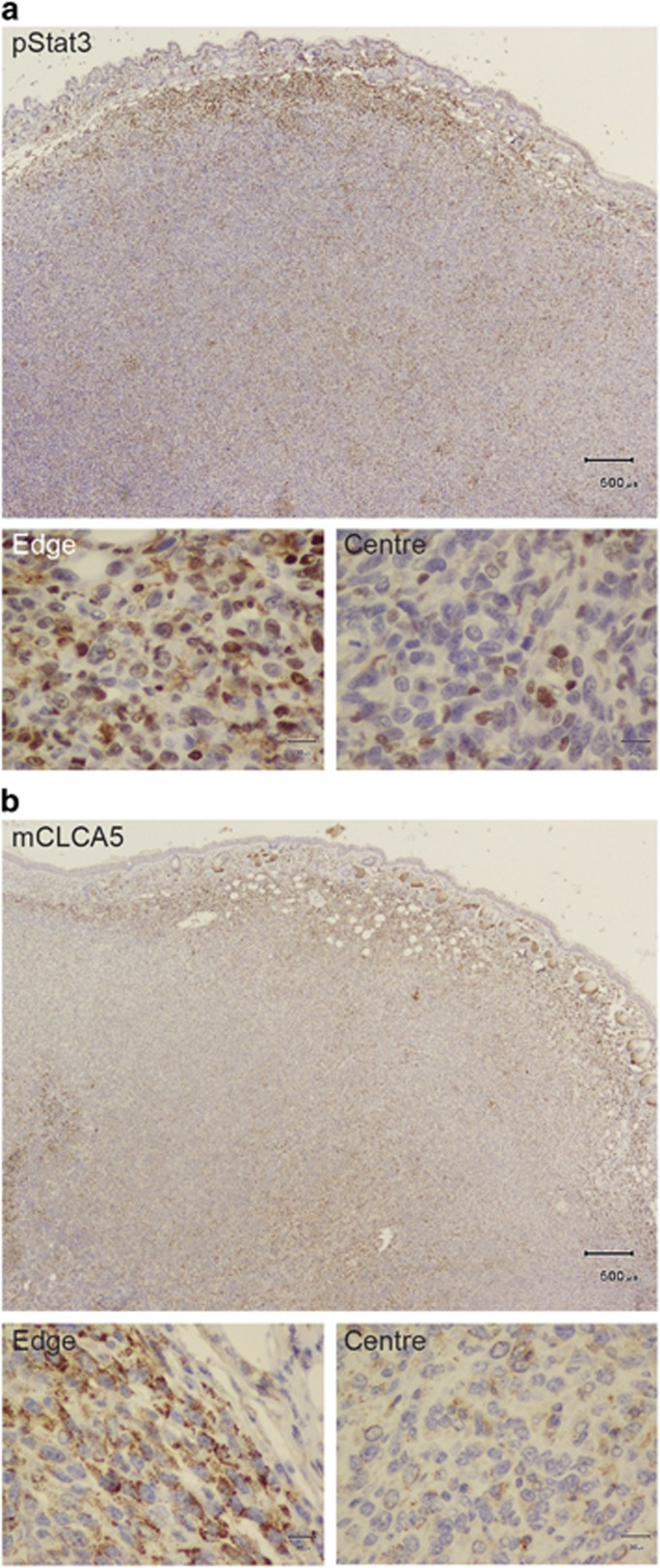
Distinct cell populations express mCLCA5 and nuclear Stat3 at the invasive edge of tumours resulting from implantation of 4T1 cells. (**a** and **b**) Immunohistochemical staining for pStat3 (**a**) and mCLCA5 (**b**) on adjacent sections of orthotopic tumours derived from implantation of 4T1 cells. Haematoxylin counterstain. Scale bar=500 *μ*m. The micrographs at higher magnification show representative staining from the edge and centre of the mass as indicated. Scale bar=30 *μ*m

Implantation of 4T1 cells into the mammary fat pad of syngeneic mice resulted in the development of fast growing tumours comprising pleomorphic cells with a high mitotic rate as previously described^31, 32^ (Figures 5a–c). pStat3 was upregulated in tumours derived from implantation of 4T1 cells, compared with 4T1 cells maintained *in vitro*, in which pStat3 levels are low. Unexpectedly, we discovered that a higher level of pStat3 accompanied a higher level of mCLCA5 expression (Figure 5d). Interestingly, the majority of nuclear pStat3 expression was seen at the invasive edge of the tumour, where cells with morphology consistent with tumour cells, fibroblasts and immune cells, all exhibited nuclear pStat3 localization (Figure 6a).^33^ The subgross pattern of localization of mCLCA5 was strikingly similar: punctate cytoplasmic mCLCA5 expression was present predominantly at the edge of the mass (Figure 6b). Granular or punctate cytoplasmic staining could be consistent with the expression of a protein within intracellular organelles – potentially a secretory product.^15^

**Figure 7:**
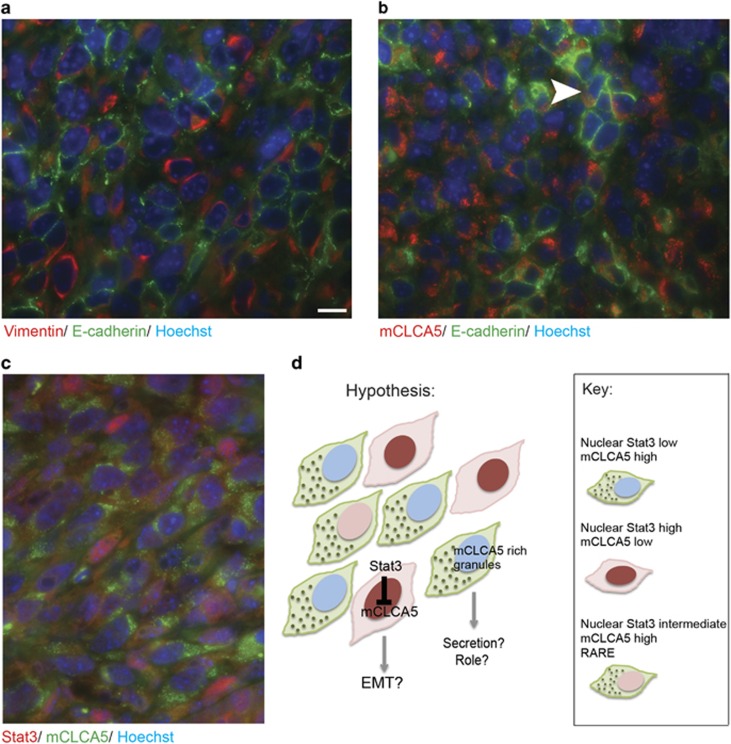
Although both mCLCA5 and pStat3 are expressed predominantly at the invasive edge of the tumour, minimal co-localization of nuclear Stat3 and cytoplasmic mCLCA5 is observed. Representative images showing the edge of orthotopic tumours derived from implantation of 4T1 cells. (**a**) Immunofluorescence staining for vimentin (red), E-cadherin (green) and DNA (Hoechst; blue). Scale bar=10 *μ*m. (**b**) Immunofluorescence staining for mCLCA5 (red), E-cadherin (green) and DNA (Hoechst; blue). Groupings of E-cadherin-positive cells express mCLCA5 (arrowhead). (**c**) Immunofluorescence staining for Stat3 (red), mCLCA5 (green) and DNA (Hoechst; blue). In each case, images are representative of results from three separate mice and are captured at the same magnification. (**d**) Graphical summary of hypothesis that subpopulations of tumour cells may express high levels of the oncogene Stat3 or mCLCA5, and that mCLCA5 may be secreted

The tumours resulting from implantation of the 4T1 cells were morphologically heterogeneous, with abundant expression of vimentin (Supplementary Figure 3). Cells expressing E-cadherin and vimentin were admixed at the invasive front, with multifocal apparent co-expression (Figure 7a and Supplementary Figure 4A). This observation underlines the potential importance of localization of mCLCA5 to the invasive front, where cells may be assuming more mesenchymal characteristics. As 4T1 cells have been previously demonstrated to express modest amounts of vimentin and robust levels of E-cadherin *in vitro*,^34^ expression of both proteins *in vivo* was perhaps unsurprising, given the aggressive phenotype of the tumours. Although the cellular heterogeneity and apparent co-expression of vimentin and E-cadherin by the 4T1 cells precluded definitive identification, by co-staining, of the mCLCA5 expressing cells as 4T1 cells, we identified E-cadherin and mCLCA5 dual-positive cells that we considered likely to be 4T1 cells (Figure 7b and Supplementary Figure 4B).

Using immunofluorescence to further characterize the cellular populations expressing mCLCA5 and nuclear Stat3 (as a surrogate read-out of Stat3 activity), we identified at least two different cell populations at the edge of the tumour (Figure 7c and Supplementary Figure 4C). Strikingly, the majority of cells either exhibited intense nuclear staining for Stat3 or strong punctate cytoplasmic staining for mCLCA5, but very few cells were identified that co-expressed mCLCA5 in the presence of nuclear Stat3. Although some of the cells expressing Stat3 exhibited morphology consistent with stromal cells or tumour-associated immune cells, others were consistent with neoplastic cells (Figure 7c).

We therefore suggest that in individual neoplastic cells, the negative regulation of mCLCA5 by pStat3 remains functional. Although both mCLCA5 and pStat3 are expressed predominantly at the invasive edge of the tumour, minimal co-localization of nuclear Stat3 and cytoplasmic mCLCA5 is observed. It seems likely that mCLCA5 expression may be predominantly in the tumour cells, whereas pStat3 nuclear localization is seen in both the tumour cells and immune and stromal compartments at the invasive front. Thus, activity of pStat3, a known breast cancer oncogene,^30, 35^ is likely to be critical to the invasive nature of the neoplastic cells at the tumour margin.

Other investigators have suggested that CLCA proteins may act as extracellular signalling molecules^17, 36^ and it is tempting to speculatively attribute such as role to mCLCA5 in this context at the invasive edge of the tumour (Figure 7).

## Discussion

We have demonstrated that mCLCA1 and mCLCA2 are profoundly downregulated in the absence of mammary epithelial Stat3 signalling during post-lactational regression, suggesting a close direct or indirect relationship between Stat3 activity and expression of mCLCA1 and mCLCA2. This exciting finding warrants further investigation in the context of the function of these proteins within the mammary gland.

Furthermore, our data suggest that during mammary gland regression, mCLCA5 expression is partially suppressed by epithelial Stat3 activity. Given the pro-tumourigenic nature of the involution mammary microenvironment, this is an important finding. However, data from orthotopic tumours derived from implantation of 4T1 cells into mice suggest that the relationship between mCLCA5 and Stat3 activity may be complex, with separate populations of cells expressing high levels of mCLCA5 or transcriptionally active (nuclear) Stat3. This suggests that Stat3 retains a suppressive effect on mCLCA5 expression in 4T1 tumour cells. It is in accordance with the findings of others, and therefore tempting to speculate, that downregulation of mCLCA5 in the presence of cells expressing Stat3 activity reflects an invasive phenotype in these cells and potential epithelial–mesenchymal transition.^16, 22, 24^

However, our data also raise the question of the role of the observed mCLCA5 expression at the invasive front. One interpretation is that mCLCA5 expression at the invasive edge may be beneficial to tumour growth with mCLCA5 acting in a non-cell autonomous manner to augment tumour cell proliferation.^37^ However, in this context it is important to note the compelling data demonstrating that re-expression of mCLCA5 in 4T1 cells inhibits proliferation^22^ and that loss of hCLCA2 expression in human MCF10A cells augments proliferation of the cells,^24^ whereas re-expression elicits a reduction in tumour cell growth.^19^ In the *in vivo* 4T1 murine model described in our study, cellular behaviour may be influenced by the presence of subpopulations of neoplastic cells in which either Stat3 activity suppresses mCLCA5 expression, or mCLCA5 is expressed in the absence of Stat3 activity. The influence of the immune cell compartment also needs to be considered particularly given that the 4T1 model involves introduction of syngeneic tumour cells into immune-competent mice. It is possible that mCLCA5 secretion is stimulated in host cells, such as stromal components or immune cells, in an attempt to negatively regulate tumour growth. Currently, the role of mCLCA5 at the invasive front of the tumour is unclear. Future work will require development of inducible models of mCLCA5 expression and/or Stat3 activity *in vivo* in different cell types to understand the relationship between the key oncogene Stat3 and the intriguing factor mCLCA5.

Although in normal mammary gland, Stat3 regulates cell death, breast cancer cells frequently become addicted to Stat3 and require Stat3 activity for survival. Our data demonstrate that Stat3 is a negative regulator of mCLCA5 during mammary gland involution, and that in tumourigenesis in an immune-competent murine model, mCLCA5 expression is suppressed in cells with high levels of nuclear Stat3. Stat3 appears to be a cell autonomous negative regulator of this protein.

## Materials and Methods

### Cell culture

Maintenance and differentiation of KIM-2 cells and instigation of hormone withdrawal, were as previously described.^28^ Human LIF (a generous gift from Dr Jennifer Nichols) or recombinant mouse OSM (495-MO, R&D Systems, Abingdon, UK) were used at a final concentration of 10 or 20 ng/ml (LIF), or 25 ng/ ml (OSM). For imaging, KIM-2 cells were differentiated in 35 mm diameter glass bottom dishes (81158, ibidi, Planegg, Martinsried, Germany). Murine 4T1 cells were purchased from ATCC (Teddington, Middlesex, UK) and were maintained according to ATCC protocols.

### RT-PCR and qRT-PCR

Standard protocols were followed.^38^ The primer sequences utilized are detailed in Supplementary Table 1.

### Immunoblotting

Standard protocols were followed.^38^ Antibodies employed were: anti-phospho-Stat3 (1:1000; Tyr705: #9131), anti-Stat3 (1:1000; #12640), anti-GAPDH (1:5000; #5174) (all Cell Signaling Technology, Danvers, MA, USA), and anti-alpha-tubulin (1:5000; AB6160, Abcam, Cambridge, UK).

### Animal husbandry

*Stat3*^*fl/fl*^; *BLG-Cre* mice^39, 40^ were utilized and involution induced as previously described.^9^ All animals were treated according to the local ethical committee and the UK Home Office guidelines.

### 4T1 tumour model

A final volume of 16 *μ*l containing 1 × 10^5^ 4T1 cells with matrigel (basement membrane matrix growth factor reduced; BD 354230; BD Biosciences, Erembodegem, Belgium) was injected into the right- or left-fourth mammary gland fat pad of ~12-week-old virgin Balb/c mice under anaesthesia and analgesia.^41^ Tumour development was monitored, mice were killed and tumours were harvested at day 25 post injection. Tumour tissue (lacking normal tissue margins) was collected for RNA and protein extraction. Tissue for histological sectioning was dissected. Formalin fixation, histological sectioning and staining followed routine protocols.

### Immunostaining

KIM-2 cells in glass bottom dishes were fixed for 10 min in ice cold methanol and immunofluorescence staining for E-cadherin (1:400; #3195; Cell Signaling Technology) was carried out following a standard protocol.

Paraffin embedded tumour specimens were prepared as three micron sections on positively charged slides (Snowcoat; Surgipath Europe Ltd, Peterborough, UK). Immunohistochemical staining for mCLCA5 (1:500; sc99224; Santa Cruz Biotechnology, Dallas, TX, USA), vimentin (1:500; #5741) and murine pStat3 (1:100; Tyr705: #9145) (both Cell Signaling Technology) was carried out using a routine protocol employing an automated immunohistochemistry system (Dako Autostainer; Dako, Ely, UK).

Immunofluoresence staining for E-cadherin (1:500; 610182, BD Biosciences, Oxford Science Park, Oxford, UK), mCLCA5 (1:100; sc99224; Santa Cruz Biotechnology), vimentin (1:200; #5741) and murine total Stat3 (1:100; #9139) (both Cell Signaling Technology, Danvers, MA, USA) was carried out following de-paraffinization and antigen retrieval for 20 min at 90 °C using Dako Envision Flex Target Antigen Retrieval Solution High pH in a PT Link and Pre-Treatment Module for Tissue Specimens (both Dako). For Fc receptor blocking prior to staining for murine total Stat3 (murine monoclonal antibody), anti-mouse CD16/CD32 clone 93 was utilized (1:500; 14-0161-85, eBioscience Ltd, Cheshire, UK). Isotype- and species-matched immunoglobulins were used as a negative control.

## Abbreviations

CLCA: chloride channel regulator, calcium-activated, also known as chloride channel accessory protein
LIF: leukaemia inhibitory factor
OSM: oncostatin M
Stat3: signal transducer and activator of transcription 3
