# Supplementary material for: Stat3 modulates chloride channel accessory protein expression in normal and neoplastic mammary tissue
Source: Cell Death Dis. 2016 Oct 6;7(10):e2398–. doi: 10.1038/cddis.2016.302 (PMC5133972; doi:10.1038/cddis.2016.302)
Supplement: Supplementary Information [file cddis2016302x1.docx]

**Supplementary Table 1**

Details primer sequences utilized in this study.

**Supplementary Figure 1. Differentiated mammary epithelial KIM-2 cells do not upregulate pStat3 expression upon stimulation with LIF.** Western blot analysis of phosphorylated Stat3 (pStat3) and GAPDH expression in differentiated KIM-2 cells stimulated with either vehicle or 20 ng/ml LIF. Four (vehicle treated) and three (LIF treated) biological repeats are shown. Data is representative of four (vehicle) and seven (LIF treated) biological repeats.

**Supplementary Figure 2. Representative images of negative control sections to accompany Figure 4.** Sections were stained with species-matched and isotype-matched control serum replacing the primary antibody mCLCA5. Scale bar = 50 μm (A) and 10 μm (B). Images are representative of sections from five mice (control n = 2; Stat3 KO n = 3).

**Supplementary Figure 3. Orthotopic tumours derived from implantation of 4T1 cells are composed of cells expressing high levels of vimentin.** Immunohistochemical staining for vimentin on sections from orthotopic tumours derived from implantation of 4T1 cells. Haematoxylin counterstain. Scale bar = 500 μm (A) and 30 μm (B). Arrowhead indicates a mitotic figure. Images are representative of sections from three mice.

**Supplementary Figure 4. Representative images of negative control sections to accompany Figure 7.** Sections were stained with species-matched and isotype-matched control serum replacing the primary antibody mCLCA5. Scale bar = 10 μm. Images are representative of sections from three mice in each case.
