# Supplementary material for: Stat3 modulates chloride channel accessory protein expression in normal and neoplastic mammary tissue
Source: Cell Death Dis. 2016 Oct 6;7(10):e2398–. doi: 10.1038/cddis.2016.302 (PMC5133972; doi:10.1038/cddis.2016.302)
Supplement: Supplementary Table 1 [file cddis2016302x2.doc]

**Table S1.** Primers used for quantitative RT–PCR analysis

| **Primer name** | **Sequence** |
| --- | --- |
| Murine β-casein | Fwd: CATTTACTGTATCCTCTGAGACT  Rev: TGCAGCTGTCCCATGAGATT |
| Murine CLCA1 | Fwd: GTGGACCAGCCTTTCTACATGT  Rev: TGTGACACAGTTGCCTCTCTCA |
| Murine CLCA2 | Fwd: GGACCGGCCTTTCTACATTTCT  Rev: CACACAGCTGCCTCTCTGACA |
| Murine CLCA5 | Fwd: ACGTGAATGGACGGAATGAGA  Rev: CAGTCTTCATGGGGGCAGAG |
| Murine Cyclophilin a | Fwd: CCTTGGGCCGCGTCTCCTT  Rev: CACCCTGGCACATGAATCCTG |
