# Supplementary figures and images for: Stat3 modulates chloride channel accessory protein expression in normal and neoplastic mammary tissue
Source: Cell Death Dis. 2016 Oct 6;7(10):e2398–. doi: 10.1038/cddis.2016.302 (PMC5133972; doi:10.1038/cddis.2016.302)

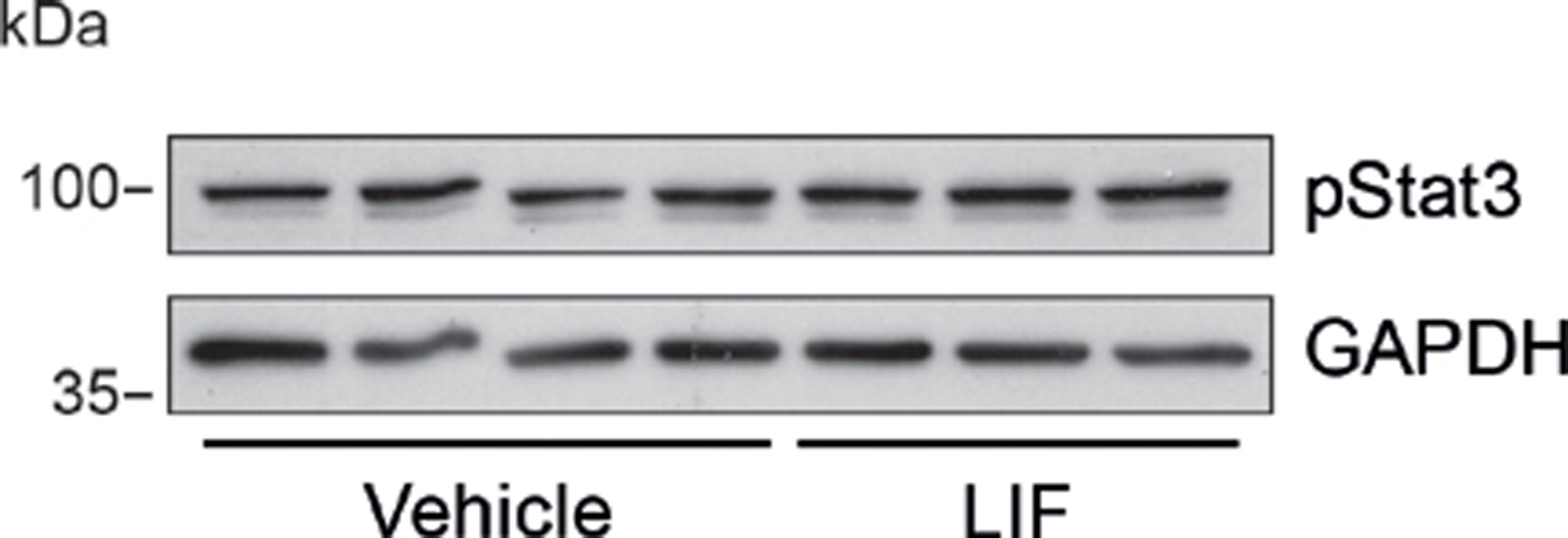

Supplement: Supplementary Figure 1 [file cddis2016302x3.tif]

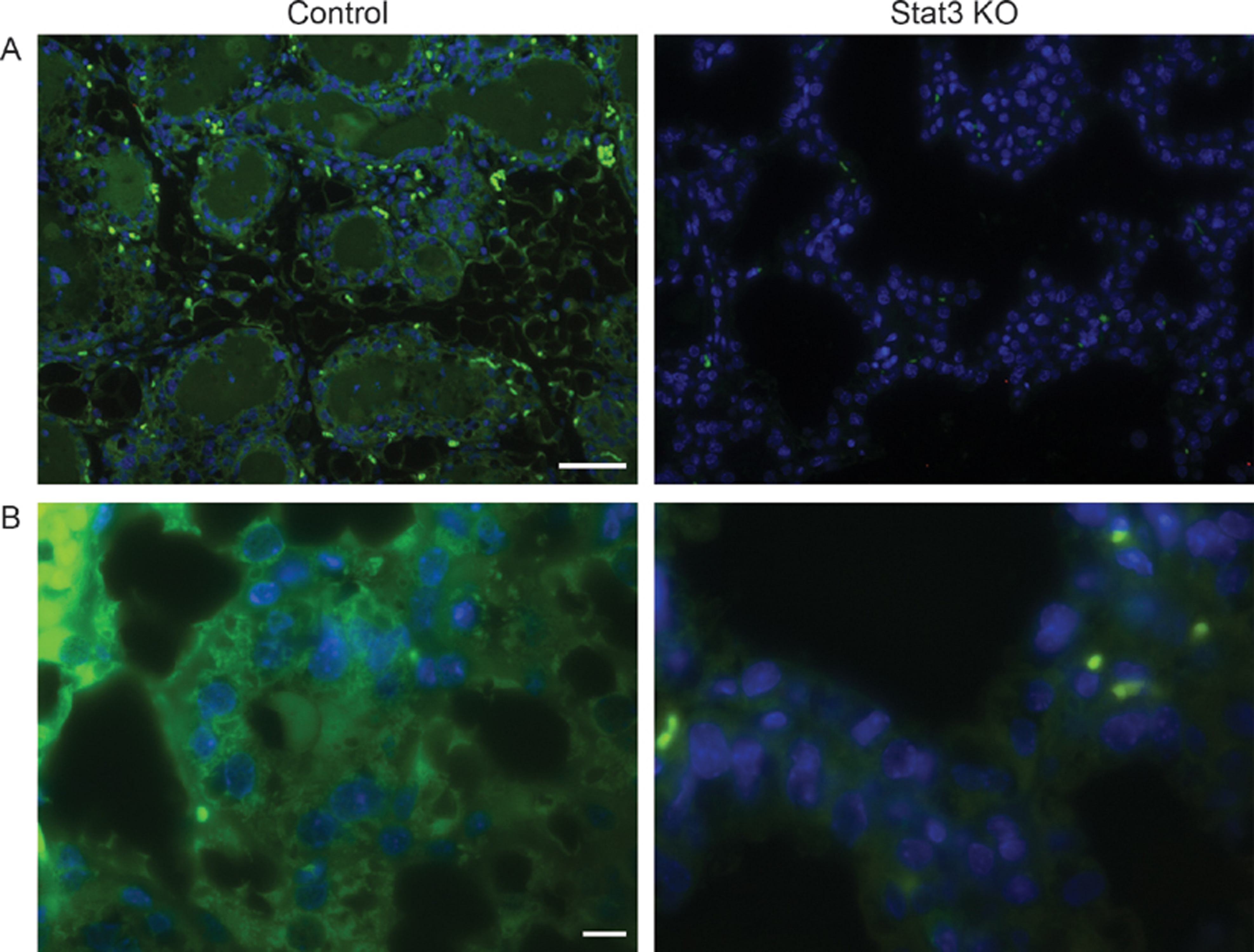

Supplement: Supplementary Figure 2 [file cddis2016302x4.tif]

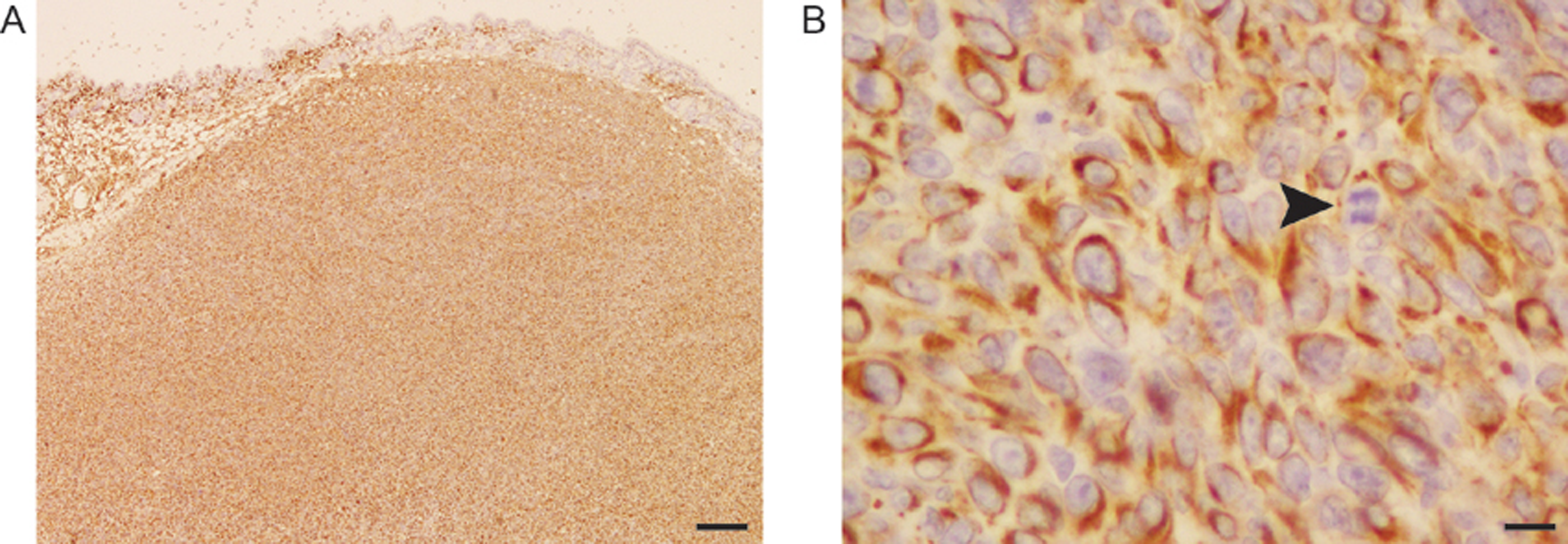

Supplement: Supplementary Figure 3 [file cddis2016302x5.tif]

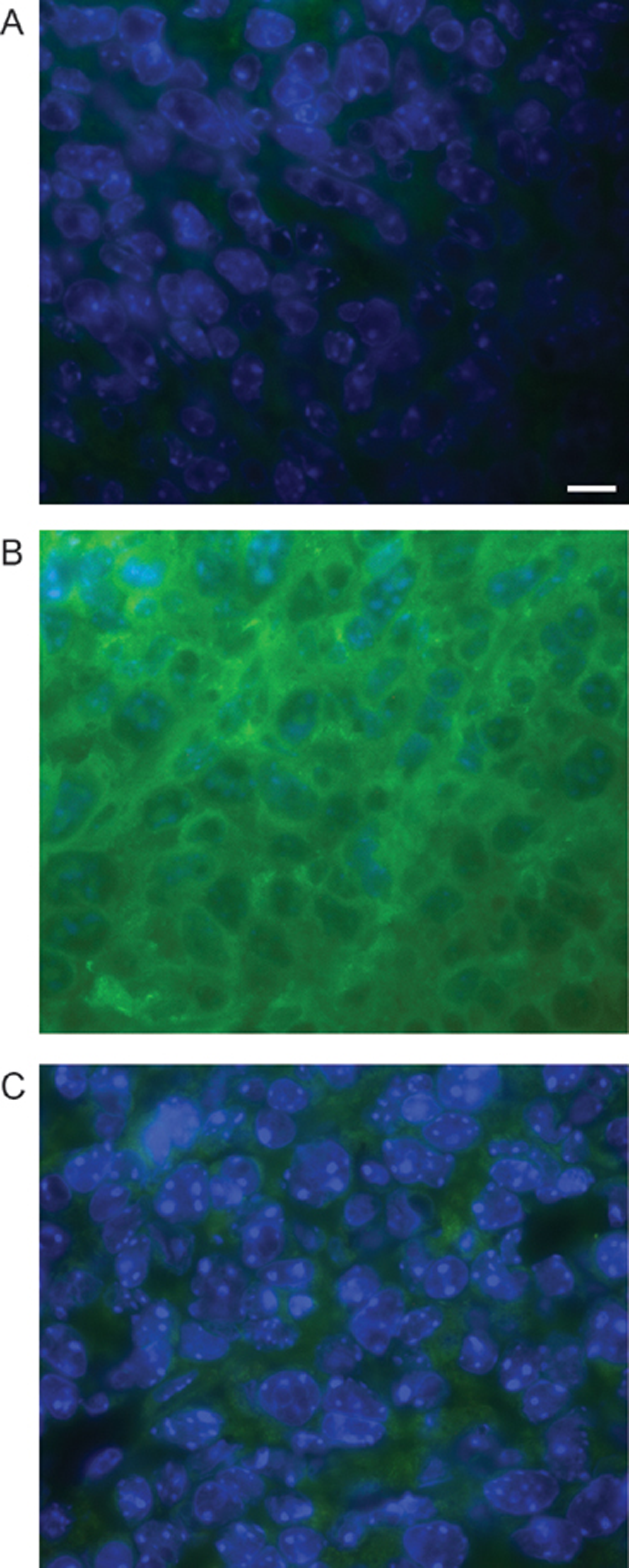

Supplement: Supplementary Figure 4 [file cddis2016302x6.tif]
